# An In Vitro Analysis on Polyurethane Foam Blocks of the Insertion Torque (IT) Values, Removal Torque Values (RTVs), and Resonance Frequency Analysis (RFA) Values in Tapered and Cylindrical Implants

**DOI:** 10.3390/ijerph18179238

**Published:** 2021-09-01

**Authors:** Luca Comuzzi, Margherita Tumedei, Camillo D’Arcangelo, Adriano Piattelli, Giovanna Iezzi

**Affiliations:** 1 Independent Researcher, Via Raffaello 36/a, 31020 San Vendemiano, TV, Italy; luca.comuzzi@gmail.com; 2 Department of Medical, Oral and Biotechnological Sciences, University “G. D’Annunzio” of Chieti-Pescara, Via dei Vestini 31, 66100 Chieti, Italy; camillo.darcangelo@unich.it (C.D.); apiattelli@unich.it (A.P.); gio.iezzi@unich.it (G.I.); 3 Biomaterials Engineering, Catholic University of San Antonio de Murcia (UCAM), Av. de los Jerónimos, 135, 30107 Guadalupe, Murcia, Spain; 4 Fondazione Villaserena per la Ricerca, Via Leonardo Petruzzi 42, 65013 Città Sant’Angelo, PE, Italy; 5 Casa di Cura Villa Serena del Dott. L. Petruzzi, Via Leonardo Petruzzi 42, 65013 Città Sant’Angelo, PE, Italy

**Keywords:** primary stability, dental implant, endosseous implants, polyurethane study, artificial bone

## Abstract

Background: Several different dental implant microgeometries have been investigated in the literature for use in low-density bone sites. The polyurethane solid rigid blocks represent an optimal in vitro study model for dental implants, because their composition is characterized by symmetrical linear chains of monomers of hexa-methylene sequences producing a self-polymerization process. The aim of the present investigation was to evaluate the primary stability of cylindrical and tapered implants positioned into low-density polyurethane solid rigid blocks. Materials and Methods: Two different macrogeometries, cylindrical (4 mm diameter and 10 mm length) and tapered dental implants (4.20 mm diameter and 10 mm length), were investigated in the present study. The implants were inserted into 10 PCF and 20 PCF polyurethane blocks, with and without an additional cortical layer. The insertion torque (IT) values, the removal torque values (RTVs), and the resonance frequency analysis (RFA) values were measured and recorded. Results: A total of 80 sites were tested, and a significant increased primary stability (PS) was detected in favour of tapered dental implants when compared to cylindrical implants in all experimental conditions (*p* < 0.05). Higher IT, RT, and RFA values were measured in tapered implants in 10 and 20 PCF polyurethane blocks, both with and without the additional cortical layer. Conclusions: Both implants showed sufficient primary stability in poor density substrates, while, on the other hand, the tapered microgeometry showed characteristics that could also lead to clinical application in low-density posterior maxillary sites, even with a drastically decreased bone cortical component.

## 1. Introduction

The attainment of optimal primary stability (PS) is an essential prerequisite for the long-term success of dental implants [1,2,3,4,5,6,7,8,9]. A good description of PS could be a lack of motion of the implant soon after placement into the bone site [8,10]. PS is produced by the biomechanical interaction between the implant surface and the peri-implant osseous tissues [4]. This interaction results from the differences between the host bone and the implant diameter [4]. For example, a 10% underpreparation of the receiving site has been reported to increase PS [11]. A low PS has been associated with a higher possibility of implant failure, while higher PS should guarantee a higher percentage of mineralized bone at the implant interface [8]. In D1, D2, and D3 bone qualities, an apparent implant failure rate of 3% has been reported, whereas in D4 bone quality, this percentage has been reported to increase to 35% [8]. Moreover, a high PS seems to indicate a very good secondary stability [8]. PS has been related to implant macrodesign, to implant surface characteristics, to the surgical technique used, and to the quality and quantity of the peri-implant surrounding bone [2,10,12,13,14,15]. In fact, in literature, it has been reported that the application of manual bone spreaders is able to preserve a significant quantity of surrounding bone with a sensible increase in the implant primary stability [15]. Important characteristics of the implant design are related to the length, diameter, shape, form, and pitch of the threads [1,2,7,8,11,12,16,17]. An optimal implant macrostructure should produce a balance between compression and traction forces, trying to reduce deleterious shear stresses [10]. Insertion torque (IT) and removal torque (RT) values provide useful information about the biomechanical occurrences at the implant–bone interface [12,13,15]. IT can be defined as the counteraction to the rotation impressed on the implant axis during insertion. Different implant shapes have been related to different IT values and different implant PS [12]. The polyurethane solid rigid block has been proposed as an artificial substitute to simulate in vitro the mechanical behaviour of the bone tissue for dental implant positioning [18,19,20,21,22,23]. This material has the advantage of a homogeneous structure, and an additional dense layer can be added to simulate the cortical component of the native jawbone anatomy in the posterior maxilla [24,25,26]. The symmetrical linear chains of polyurethane monomers, provided by hexa-methylene sequences, results in an increased level of ordered and very strong superstructures, associated with the high magnitude of crystallinity of the polyurethane phase, and a large self-assembling process of linear hard components [27].

The aim of the present study was an in vitro evaluation, in polyurethane blocks of different densities, of the IT, RT, and of resonance frequency analysis (RFA) values of tapered and cylindrical implants. The null hypothesis was that there was no difference between the tapered and cylindrical implants.

## 2. Materials and Methods

In the present study, 4.2 mm diameter and 10 mm length tapered implants (Is-Four) and 4.0 mm diameter and 10 mm length cylindrical implants (Cyroth) (AoN Implants S.r.l., Grisignano di Zocco, Vicenza, Italy) were analysed in 10 and 20 PCF polyurethane blocks, with or without a 1-mm 30 PCF cortical sheet, for a total of 80 sites. Insertion torque (IT), removal torque (RT), and resonance frequency analysis (RFA) were recorded. A 0.2 mm underpreparation was done for tapered implants, while a 0.6 mm underpreparation was done for cylindrical implants in both polyurethane blocks of 10 and 20 PCF.

### 2.1. Implants Characteristics 

Two different dental implant macrogeometries were tested in the present investigation:

- Group I: Cylindrical implants (Cyroth, AoN Implants, Grisignano di Zocco, VI, Italy) with 4 mm diameter and 10 mm length;
- Group II: Tapered implants (Is-Four, AoN Implants, Grisignano di Zocco, VI, Italy) with 4.20 mm diameter and 10 mm length.

**Figure 1:**
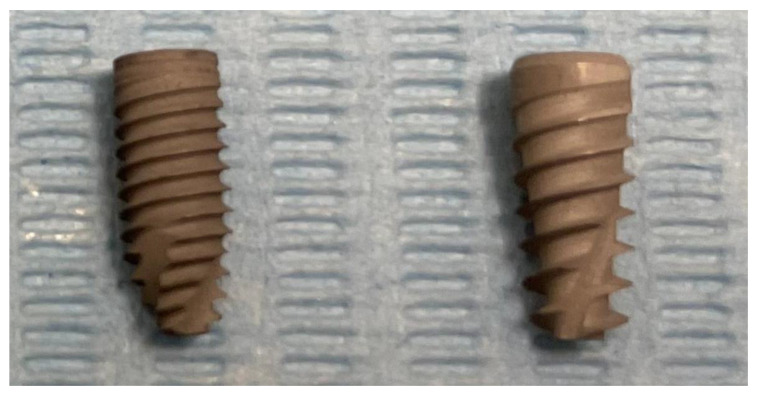
Details of the cylindrical and tapered implants tested in the present investigation.

The tapered implant was characterized by a Cone Morse self-locking prosthetic connection—an aggressive thread profile able to improve the primary stability in low-density bone and a self-tapping apex (Figure 1).

The cylindrical implant was characterized by a Cone Morse self-locking prosthetic connection, and a conical apex that seemed to be optimal for an underpreparation protocol. The self-tapping parallel wall profile appeared to be very versatile, and particularly suitable for dense bone. Both implants were characterized by a surface treatment obtained by a dual acid-etching process generating a microtopographic characteristic.

### 2.2. Polyurethane Foam Blocks

A total of 80 implants were tested in the present in vitro investigation: 40 implants for each study group, which were tested on different densities of solid rigid polyurethane blocks (SawBones H, Pacific Research Laboratories Inc, Vashon, WA, USA):

- 10 pounds per cubic foot (PCF) polyurethane density without a cortical layer;
- 10 pounds per cubic foot (PCF) polyurethane density with a 1-mm cortical layer (30 PCF);
- 20 pounds per cubic foot (PCF) polyurethane density without a cortical layer;
- 20 pounds per cubic foot (PCF) polyurethane density with a 1-mm cortical layer (30 PCF).

According to the Misch classification, the 10 PCF polyurethane block simulated the D4 bone density and the 20 PCF polyurethane block was similar to D2 human bone density [28].

### 2.3. Implant Site Drilling Protocol

The implant site preparation procedure was performed in accordance with the manufacturer’s protocol (AoN Implants, Grisignano di Zocco VI, Italy)**.** The drills sequence for the tapered implant was as follows: lance drill (800 rpm and 30 Ncm), 2.2 mm Ø drill (800 rpm and 30 Ncm), 2.8 mm Ø drill (500 rpm and 30 Ncm), 4.2 conical dedicated Ø drill at 100 rpm and 30 Ncm. The drills sequence for cylindrical implant was as follows: lance drill (800 rpm 30 Ncm), 2.2 mm Ø drill (800 rpm and 30 Ncm), 2.8 mm Ø drill (500 rpm and 30 Ncm), 3.4 mm Ø drill at 100 rpm and 30 Ncm. The implant positioning was performed at 30 rpm and 35 Ncm.

### 2.4. Primary Stability Assessment

The insertion torque was assessed manually, measured by an electronic torque meter Implantork (ANDILOG, Pinellas Park, FL, USA), considering the last 1 mm from the final position of the implant into the preparation site. The device is provided by an auto-calibrated built-in digital dental implant torque wrench device.

**Figure 2:**
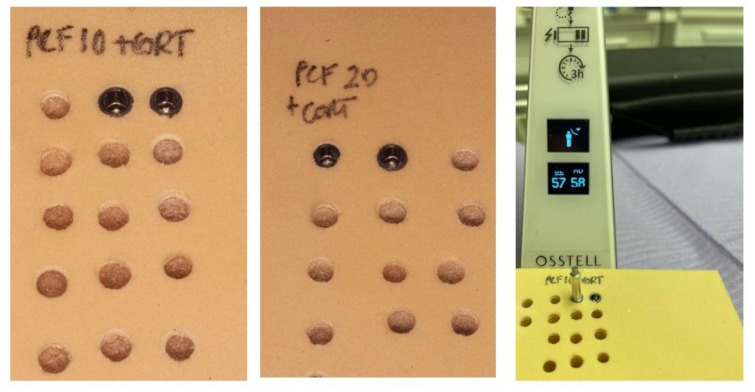
Details of the cylindrical and tapered implants positioned into polyurethane solid rigid blocks. Left: Implant devices inserted into 10 PCF + 1 mm cortical layer block. Centre: Implant devices inserted into 20 PCF + 1 mm cortical layer block. Right: Primary stability evaluated through the resonance frequency analysis device.

The resonance frequency analysis (RFA) was assessed by an electronic device (Type 78, Osstel, Columbia, MD, USA), after the screw positioning (Figure 2). The values were registered in accordance with the implant stability quotient score (ISQ). The RFA assessment was performed two times for each implant. The removal torque value (RTV) was assessed by an electronic torque meter, considering the highest peak necessary to produce the unscrewing of the implant fixture from the polyurethane block (Figure 2). 

### 2.5. Statistical Analysis

The study data were registered and analysed by a special data form produced by the statistical software package GraphPad 6 (Prism, San Diego, CA, USA). The normality distribution of the study data was evaluated by the Shapiro–Wilk test. Potential differences in study variables between the study groups was evaluated by the one-way ANOVA test followed by the Tukey post-hoc test. The level of significance was set at *p* < 0.05. 

The sample size was calculated in accordance to the findings of a previous study [20], applying an alpha error of 0.05, an effect size of 0.45, and power (1-beta) of 0.80. The minimum specimens calculated for all experimental conditions were 80 implants.

## 3. Results

**Figure 3:**
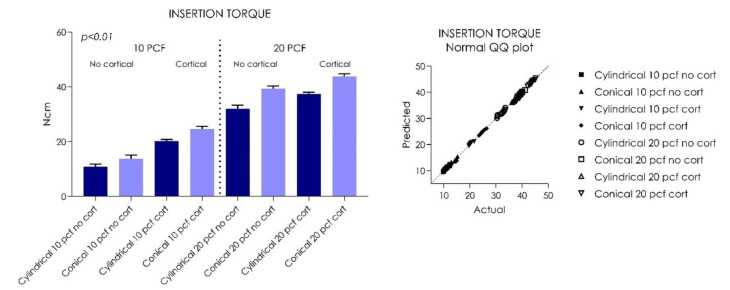
Graph and QQ plot values distribution showing the insertion torque (IT) values of the tested cylindrical and tapered implants.

**Figure 4:**
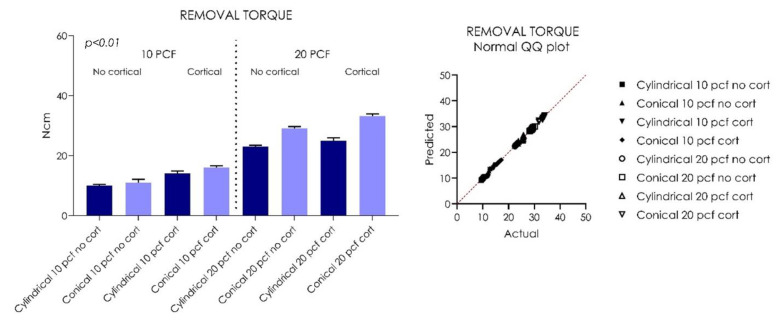
Graph and QQ plot values distribution showing the removal torque (RT) values of the tested cylindrical and tapered implants.

**Figure 5:**
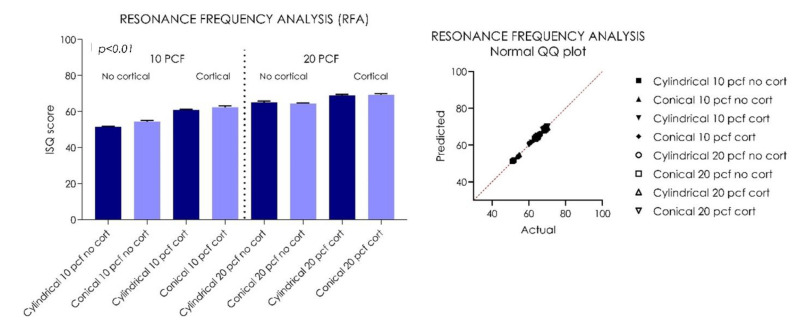
Graph and QQ plot values distribution showing the resonance frequency analysis (RFA) values of the tested cylindrical and tapered implants.

The tapered implants (Group I) showed a significantly higher insertion torque (IT) compared to the cylindrical implants in all situations, ranging from a minimum of 12 Ncm in 10 PCF blocks without the cortical layer to a maximum of 43.4 Ncm in 20 PCF blocks with 1 mm of cortical layer (Table 1, Figure 3). The gap between the tapered implants (Group II) and the cylindrical implants remained fairly constant as the bone consistency varied (*p* < 0.01). In all polyurethane consistencies, the presence of a 1-mm cortical layer was able to increase the IT, RT, and RFA means for both tapered and cylindrical implants (*p* < 0.01) (Table 1, Table 2, Table 3, Figure 3, Figure 4, Figure 5).However, in increased PCF densities (20 PCF), these tapered implants led to a high IT, with greater stress on the material. The RT values were lower for both types of implants for both PCF densities. Better performance was found in RT values for the cylindrical implants, probably due to their parallel-walled macrogeometry. All implants, in all experimental conditions, presented very good stability in the polyurethane, with ISQ values varying from about 50 to about 70, with the only exception being cylindrical implants inserted in 10 PCF blocks, without the presence of the cortical sheet. The higher values (70 ISQ) were recorded in tapered implants in 20 PCF blocks, with 1 mm of 30 PCF cortical sheet; the lowest values were recorded in cylindrical implants without the presence of the cortical sheet (48–50 ISQ). 

### 3.1. 10 PCF Artificial Bone Density

Both cylindrical and tapered implants showed good stability in low bone consistencies (10 PCF) without the presence of a cortical layer, with Group I showing a mean of 10.92 ± 0.85 Ncm, and Group II showing a mean of 13.80 ± 1.2. The mean RTV of Group I implants was 9.98 ± 0.4 Ncm, and that of Group II was 10.98 ± 1.1 Ncm (*p* < 0.01). 

The resonance frequency analysis (RFA) values of Group I implants was 51.55 ± 0.81 Ncm, and that of Group II was 54.45 ± 0.4 Ncm in 10 PCF density blocks without the cortical component (*p* < 0.01) (Table 1, Table 2, Table 3, Figure 3, Figure 4, Figure 5). In 10 PCF blocks with a 1-mm cortical layer, Group II showed higher IT, RT, and RFA values compared to Group I (*p* < 0.01), with Group I showing a mean of 20.23 ± 0.58 Ncm, and Group II showing a mean of 24.62 ± 0.89 Ncm. The mean RTV of Group I implants was 14.09 ± 0.81 Ncm, and that of Group II was 16.01 ± 0.6 Ncm (*p* < 0.01). The RFA value of Group I implants was 60.85 ± 0.47 Ncm, and that of Group II was 62.35 ± 0.70 Ncm in 10 PCF density blocks with the cortical component (*p* < 0.01) (Table 1, Table 2, Table 3, Figure 3, Figure 4, Figure 5).

### 3.2. 20 PCF Artificial Bone Density

In 20 PCF density blocks, both implants showed a significant increase in primary stability parameters (*p* < 0.05). In 20 PCF blocks without the cortical component, Group I showed a mean IT of 32.07 ± 1.19 Ncm, and Group II showed a mean of 39.37 ± 0.9 Ncm. The mean RTV of Group I implants was 23.07 ± 0.4 Ncm, and that of Group II was 29.12 ± 0.57 Ncm (*p* < 0.01) (Table 1, Table 2, Table 3, Figure 3, Figure 4, Figure 5).

The RFA value of Group I implants was 65.00 ± 0.84 Ncm, while that of Group II was 64.35 ± 0.47 Ncm in 20 PCF density blocks without the cortical component (*p* < 0.01). In 20 PCF blocks with a 1-mm cortical layer, Group I showed a mean of 37.34 ± 0.69 Ncm and Group II showed a mean of 43.80 ± 0.92 Ncm. The mean RTV of Group I implants was 25.03 ± 0.92 Ncm, and that of Group II was 33.19 ± 0.75 Ncm (*p* < 0.01). The RFA value of Group I implants was 68.85 ±0.62 Ncm, and that of Group II was 69.20 ± 0.78 Ncm in 20 PCF density blocks with the cortical component (*p* < 0.01) (Figure 3, Figure 4, Figure 5).

## 4. Discussion

Based on the findings of the present in vitro investigation, the null hypothesis was rejected, which was in agreement with other previous studies [20,21]. A still open question is in regard to which implant macrogeometry or shape will have the most relevant influence on the establishment of an optimal PS [17]. The two main implant configurations present on the market are cylindrical (or parallel-walled) and tapered (also called conical). The results of the present study showed that improved implant performance was found in higher polyurethane densities, as already reported in the literature, where a direct relationship between PS and bone density was found [4,7,11,28,29,30,31]. Moreover, the polyurethane solid rigid blocks are able to avoid the local and anatomical variability of the native bone tissues, with no mechanical alteration connected with the environmental condition, and produce a standardized model for dental implant testing [20,23,26]. On the contrary, a limitation of the polyurethane substrates is that this material does not permit a validated evaluation of other parameters, such as the drilling temperature, or histological findings, and does not provide a consistent translational comparison of the implant interface with a human bone. In accordance with these characteristics, the ASTM (American Society Testing Materials) considered polyurethane blocks to be an ideal material for the comparative testing of dental implant behaviour [23]. The polyurethane blocks are available in different microstructures and densities and are able to simulate human cancellous and cortical bone, thereby providing a standardization of the procedures without the anatomical and structural heterogenicity of native bone [23,32]. Moretti Neto et al. validated the in vitro model, reporting that a polyurethane experimental model built in a 1:1 ratio presented an appropriate modulus of elasticity to simulate bone in in vitro tests with strain gauges [33]. In the cases in which a 1-mm cortical sheet was added to the blocks, higher values were found in the present study. The results of this study also showed that the implant morphologies were well adapted to polyurethane densities similar to bone type D2 and D4. In the case of increased bone density, the cylindrical implants were able to produce less stresses and reduced pressures to the marginal bone tissue, while in lower consistencies, the tapered implants were able to improve the implant PS. Low bone densities are generally localized at the level of the posterior maxilla, areas where a tapered shape could be indicated, while in the mandible, cylindrical geometry could be preferred [28,34]. The presence of a 1-mm cortical thickness of high-density polyurethane, mimicking the cortical bone, appeared to be important for increasing the stability parameters for both implants, while, if absent, the tapered implants seemed more suitable because they guaranteed a higher stability. However, the cylindrical implants were well adapted in any situation, and should be preferred in some situations, as it is sufficient to increase the underpreparation to obtain good stability (0.3 mm underpreparation seems to be enough), with better RT values [35]. They also have other advantages because they are easier to manage while drilling, and the cylindrical shape of the prepared bone sites helps in maintaining the direction and position of the subsequent drills, without the risks of inaccuracies and over-preparation [12]. In the present study, two different underpreparation techniques was evaluated, in accordance with the main differences of the behaviours of the two different implant shapes tested, while a high influence could also be determined by the microgeometry, macrogeometry, and thread profile. In the present study, the tapered implant showed a more aggressive thread profile, with higher penetration compared to the cylindrical fixtures that require an individual adjustment of the underpreparation protocol. This aspect could represent a clinical advantage in favour of the tapered implant, which could represent a more manageable device in the presence of a very low bone density and in the case of scarce cortical bone component [2,10]. The drilling of conical implants is less accurate in maintaining position and direction, and implant stability is achieved only when the implant has reached the bottom of its position. In addition, both morphologies are characterized by threads extended to the coronal portion in order to facilitate the subcrestal insertion of the implants [18,36]. Moreover, according to the macrogeometry, the cylindrical design is ideally able to increase the contact area, with the surrounding compact tissue generating a higher bone-to-implant contact percentage if compared to the conical shape [17,37,38]. In this way, many other mechanical and technical factors are able to influence the PS, such as the positioning technique, the local bone density, and the surgical approach. Additionally, the microgeometry and the surface texture, the thread pitch, and the roughness could also significantly influence the PS [28,31,39,40,41,42,43]. In the present investigation, both of the tested implants presented a dual acid-etching process as a surface roughness treatment. The importance of the thickness of the cortical bone in obtaining a better PS has already been reported by Romanos et. al., Toyoshima et al., and Marquezan et al [11,16,31]. No significant differences were found in the results of the stability obtained with the use of tapered vs. cylindrical implants. Both implants presented good PS, mainly related to the presence of an additional cortical sheet. Apparently, there is a direct relationship between an increased IT and an increased PS [31]. However, a high IT during implant insertion could produce an adverse micromovement at the interface, with the possible formation of fibrous connective tissue, and not mineralized tissue at the implant interface [2]. In the present study, it was found that tapered implants produced a higher stress on the surrounding material in increasing polyurethane densities. 

## 5. Conclusions

The present study showed that both of the dental implant macrogeometries investigated in the present study produced sufficient primary stability when positioned in artificial bone substitutes. In all experimental conditions, the tapered dental implants showed an improved PS when positioned into low-density polyurethane blocks compared to the cylindrical macrogeometry. This fact could help clinicians in choosing an implant better suited for use in poor density bone tissue.

## Figures and Tables

**Table 1:** Summary of insertion torque (IT) values of the tested cylindrical and conical implants.

Insertion Torque	10 PCF				20 PCF			
	No cortical layer		Cortical layer		No cortical layer		Cortical layer	
	Cylindrical	Conical	Cylindrical	Conical	Cylindrical	Conical	Cylindrical	Conical
Mean	10.92	13.80	20.23	24.62	32.07	39.37	37.34	43.80
Std. Deviation	0.8522	1.244	0.5851	0.8979	1.191	0.9129	0.6963	0.9274
Lower 95% CI	10.31	12.91	19.81	23.98	31.22	38.72	36.84	43.14
Upper 95% CI	11.53	14.69	20.65	25.26	32.92	40.02	37.84	44.46

**Table 2:** Summary of the removal torque (RT) values of the tested cylindrical and conical implants.

Removal Torque	10 PCF				20 PCF			
	No cortical layer		Cortical layer		No cortical layer		Cortical layer	
	Cylindrical	Conical	Cylindrical	Conical	Cylindrical	Conical	Cylindrical	Conical
Mean	9.980	10.98	14.09	16.01	23.07	29.12	25.03	33.19
Std. Deviation	0.4077	1.121	0.8157	0.6226	0.4029	0.5731	0.9274	0.7564
Lower 95% CI	9.688	10.18	13.51	15.56	22.78	28.71	24.37	32.65
Upper 95% CI	10.27	11.78	14.67	16.46	23.36	29.53	25.69	33.73

**Table 3:** Summary of the resonance frequency analysis (RFA) values of the tested cylindrical and conical implants.

RFA	10 PCF				20 PCF			
	No cortical layer		Cortical layer		No cortical layer		Cortical layer	
	Cylindrical	Conical	Cylindrical	Conical	Cylindrical	Conical	Cylindrical	Conical
Mean	51.55	54.45	60.85	62.35	65.00	64.35	68.85	69.20
Std. Deviation	0.3689	0.4972	0.4743	0.7091	0.8498	0.4743	0.6258	0.7888
Lower 95% CI	51.29	54.09	60.51	61.84	64.39	64.01	68.40	68.64
Upper 95% CI	51.81	54.81	61.19	62.86	65.61	64.69	69.30	69.76

## Data Availability

All experimental data to support the findings of this study are available upon request from the corresponding author.
